# Electrochemical Analysis of Methanol with Nafion-Coated Copper Oxide Nanoparticles

**DOI:** 10.1155/2024/1809578

**Published:** 2024-09-28

**Authors:** Pantipa Sawatmuenwai, Keerakit Kaewket, Kamonwad Ngamchuea

**Affiliations:** School of Chemistry Institute of Science Suranaree University of Technology, 111 University Avenue, Suranaree, Muang, Nakhon Ratchasima 30000, Thailand

## Abstract

This work introduces a Nafion-coated copper(I) oxide nanoparticle electrode (Nafion/Cu_2_O/GC) designed for the electrochemical detection of methanol (CH_3_OH). The responses of the composite material toward CH_3_OH were enhanced by the selective permeation of CH_3_OH through the hydrophilic channels of the Nafion membrane in combination with the electroactivity of Cu_2_O nanoparticles. The sensor displayed a linear detection range of 0.33–100 mM CH_3_OH with a sensitivity of 0.17 *μ*A·mM^−1^ and a detection limit (3 s/m) of 0.10 mM. It exhibited excellent reproducibility with a relative standard deviation of <5%. The sensor's practical applicability was demonstrated through recovery studies on hand sanitizer samples, achieving ca. 100% recovery. The sensor was further used to elucidate CH_3_OH adsorption on activated carbon, revealing that the process conforms to the Langmuir isotherm model.

## 1. Introduction

Methanol (CH_3_OH) is a transparent and volatile liquid widely recognized for its extensive applications as a chemical feedstock, fuel, solvent, and antifreeze agent [1]. Its electrochemical characteristics are essential in several technological deployments, notably in the operation of methanol fuel cells and electrochemical flow batteries. However, methanol's potential toxicity raises significant safety concerns, particularly when contaminated in consumer products such as alcoholic drinks and hand sanitizers [2, 3], posing serious health risks upon ingestion, inhalation, or dermal contact [4]. This emphasizes the urgent need for stringent surveillance to prevent incidents of methanol poisoning. This work therefore investigates the electrochemical processes involving methanol with a particular focus on analytical applications.

Efforts to optimize the electrochemical reactions of methanol have led to the study of various electrocatalysts, including Pt [5, 6], Pd [6, 7], Ru [8], and Au [9]. In addition to noble metals, Co [10, 11] and Cu [12–14], being cheaper and more abundant materials, have shown potential to replace noble metals in methanol reactions. It has been reported that the initial step in methanol reactions involves OH bond breaking to form the surface-bound methoxy (CH_3_O∗) intermediate [15], which typically leads to the formation of formaldehyde, formic acid, or CO_2_ as the products. The presence of oxygen, hydroxyl, or water on the Cu surface alters the methanol reaction pathway, often promoting increased methoxy formation and overall electroactivities [16–18]. Due to its thermodynamic stability and surface oxygen presence, Cu_2_O has gained prominence in these studies, particularly when utilized in a nanoparticle form. These nanoparticles significantly amplify the electrocatalytic performance and increase the electroactive surface area, enhancing the overall efficiency of the reactions [14].

However, challenges in the electrochemical reactions at Cu₂O nanoparticles remain, especially regarding their charge transfer resistance, conductivity, and sensitivity, in various electrochemical environments. In addition, the aggregation of nanoparticles can hinder their performance. To address these issues, Nafion emerges as a key component in electrode materials. Formed through the polymerization of tetrafluoroethylene and perfluorinated vinyl ether [19], Nafion exhibits a negative surface charge that facilitates proton interaction, prevents the adsorption and diffusion of anionic species to the electrode surface, and most importantly, enhances the electrochemical responses of Cu_2_O [20–22]. The material exhibits outstanding ionic and proton conductivity, selective cation transport, mechanical and chemical durability, a microscopic network configuration, and antifouling characteristics. Furthermore, Nafion serves the additional function of binding to prevent Cu_2_O detachment from the electrode surface [21].

**Scheme 1:**
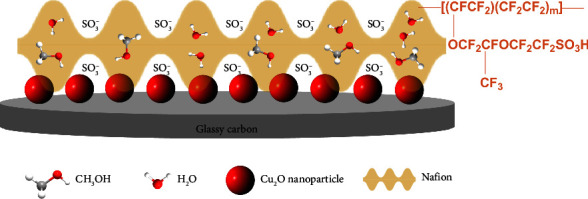
Schematic diagram showing the Nafion-coated Cu_2_O nanoparticles-modified glassy carbon electrode (Nafion/Cu_2_O/GC) for the electrochemical analysis of methanol.

This work demonstrates that the combination of Nafion and Cu_2_O nanoparticles (Scheme 1) can significantly enhance the responses to methanol, offering potential benefits in numerous electrochemical applications. The developed method serves as a promising alternative to conventional analytical methods, which often require expensive and bulky equipment, limiting their applicability to laboratory settings, such as gas chromatography [23, 24], high performance liquid chromatography [25], fluorescence [26], Raman spectroscopy [27], X-ray absorption spectroscopy [28], UV-visible spectroscopy [29], and X-ray photoelectron spectroscopy [29]. The developed sensor presents a low-cost, simple, portable, and user-friendly solution for methanol detection. Furthermore, the proposed method offers a high degree of selectivity and sensitivity, which are crucial for accurately detecting methanol in complex matrices. In addition, we will demonstrate that the developed sensor can also be applied to evaluate the methanol adsorption process, for example, on the surface of activated carbon, one of the most commonly used sorbent materials.

## 2. Experimental

### 2.1. Chemical Reagents

All chemicals were of analytical grade and were used as received without further purification: methanol (CH_3_OH, 99.8%, ANaPURE), potassium chloride (KCl, ≥99.0%, Sigma-Aldrich), copper(I) oxide (Cu_2_O, 97%, Sigma-Aldrich), Nafion (D-520, 1,1,2,2-tetrafluoro-2-((1,1,1,2,3,3-hexafluoro-3-[(1,2,2-trifluoroethenyl)oxy]propan-2-yl)oxy)ethane-1-sulfonic acid; tetrafluoroethene, 5% w/w in water and 1-propanol, Thermo Scientific Chemicals), and deionized water (ELGA LabWater, UK). Activated carbon was obtained from IRPC Public Company Limited, Thailand.

### 2.2. Characterization of Cu_2_O and Nafion-Coated Cu_2_O Nanoparticles

The morphology and size distribution of Cu_2_O nanoparticles deposited on the glassy carbon surface, both in the presence and absence of Nafion, were analyzed using a field emission scanning electron microscope (FE-SEM, 3.00 kV, Zeiss AURIGA, Germany). Powder X-ray diffraction (XRD) analysis of Cu_2_O nanoparticle powder was performed utilizing a Bruker D8 ADVANCE instrument, Germany, employing Cu-K radiation with a wavelength (*λ*) of 0.15406 nm covering 2*θ* in the range of 20°–80°. Fourier transform infrared spectroscopy (FTIR) was employed to investigate the surface functional groups of Cu_2_O, Nafion, and Nafion-coated Cu_2_O nanoparticles immobilized on a glassy carbon substrate.

### 2.3. Electrochemical Measurements

All electrochemical measurements were performed in deoxygenated aqueous solutions saturated with nitrogen gas (99.995% purity, Thai Special Gas, Thailand) enclosed within a Faraday cage at a temperature of 25°C using a PalmSens4 potentiostat (PalmSens, Netherlands) and a standard three-electrode system. The employed working electrode was a Nafion-coated copper(I) oxide nanoparticle-modified glassy carbon electrode, denoted as Nafion/Cu_2_O/GC. The glassy carbon macrodisc electrode (GC), with a diameter of 3.0 mm, was obtained from ItalSens (Netherlands). A silver/silver chloride electrode in saturated KCl (Ag/AgCl, ItalSens, Netherlands) served as the reference electrode. A platinum wire (0.5 mm diameter, 70 mm length, ItalSens) was utilized as the counter electrode. Potassium chloride at a concentration of 0.10 M was used as the supporting electrolyte.

To fabricate the Nafion/Cu_2_O/GC, the GC electrode underwent initial mechanical polishing using alumina powder (1.0, 0.3, and 0.05 µm) on MicroCloth™ soft polishing cloths (Buehler, USA). A suspension containing 1.8 mg of Cu_2_O nanopowder in 1.0 mL of deionized water was prepared and subjected to 15 minutes of sonication (at 50 W and 40 kHz). Subsequently, 5 *μ*L of this Cu_2_O nanopowder suspension was drop-casted onto the GC electrode and oven-dried at 50°C for 10 minutes under vacuum conditions (DZ-A/BC II Series, China) to form the Cu_2_O/GC electrode. The Nafion suspension was then formulated by diluting 50 *μ*L of Nafion (5% w/w, Thermo Scientific Chemicals) in 450 *μ*L of water, followed by 1 minute of sonication (at 50 W and 40 kHz). Following this, 5 *μ*L of the Nafion suspension was drop-casted onto the Cu_2_O/GC electrode and similarly dried at 50°C for 10 minutes under vacuum. The fabricated Nafion/Cu_2_O/GC electrode was finally cooled to room temperature before utilization.

### 2.4. Measurement of CH_3_OH in Hand Sanitizer Samples

The developed Nafion/Cu_2_O/GC electrode was applied for the detection of CH_3_OH in three different brands of hand sanitizers acquired from a local market in Nakhon Ratchasima, Thailand. Initially, the samples were intentionally spiked with 10 mM of CH_3_OH. To quantify CH_3_OH concentrations in the spiked samples, the standard addition method was employed. The analysis was conducted via cyclic voltammetry measurements at the Nafion/Cu_2_O/GC electrode, employing a scan rate of 50 mV·s^−1^ in the presence of 0.10 M KCl supporting electrolyte. The resulting peak currents were analyzed, and the findings were presented as a percentage recovery ± standard deviation.

### 2.5. Evaluation of CH_3_OH Adsorption on Activated Carbon

To assess the adsorption of CH_3_OH on activated carbon, 8 g of carbon was mixed with 40.0 mL of CH_3_OH solutions ranging from 0 to 100 mM. The mixture was sealed and agitated on a Labcon SPL-MP 15 orbital shaker at 25°C and 155 rpm for varying periods (0–180 minutes). Subsequently, the mixture was filtered through a Whatman filter paper with an 11 *μ*m pore size. The filtrate was collected and purged with nitrogen gas for 3 minutes. The concentration of residual CH_3_OH in the solution was quantified using a Nafion/Cu_2_O/glassy carbon electrode via cyclic voltammetry at a scan rate of 50 mV·s^−1^ in the presence of 0.10 M KCl supporting electrolyte.

## 3. Results and Discussion

Initially, the physicochemical properties of Cu_2_O and Nafion-coated Cu_2_O nanoparticles were assessed, focusing on their morphology, particle size, crystallography, surface functional groups, and, most importantly, their electrochemical behaviors. Subsequently, the sensor's analytical performance was evaluated and optimized to ensure accuracy and selectivity in CH_3_OH quantification. The sensor was then used to analyze CH_3_OH in hand sanitizer samples and to evaluate CH_3_OH adsorption on the surface of activated carbon.

### 3.1. Physicochemical Properties of Cu_2_O and Nafion-Coated Cu_2_O Nanoparticles

**Figure 1:**
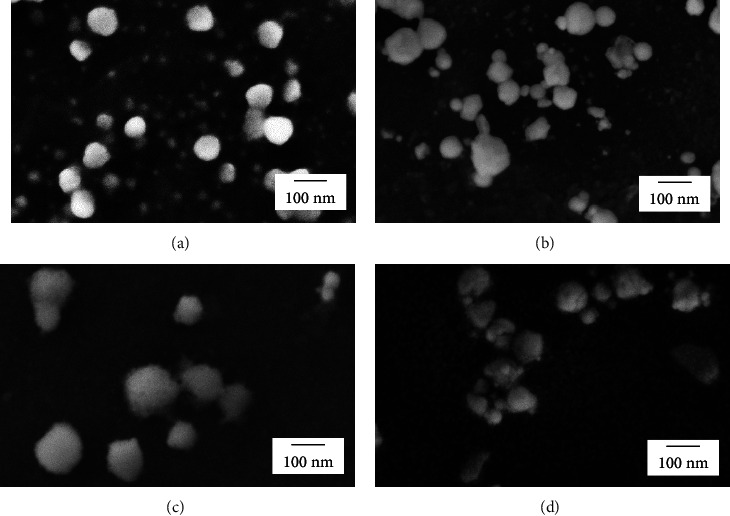
SEM images of Cu_2_O nanoparticles (a) before and (b) after voltammetric measurements of CH_3_OH, and Nafion-coated Cu_2_O nanoparticles (c) before and (d) after voltammetric measurements of CH_3_OH (*E* = 1.4 ⟶ −0.8 ⟶ 1.4 V vs. Ag/AgCl (saturated KCl)).

The scanning electron microscopic image in Figure 1(a) reveals that the Cu_2_O nanoparticles appear mostly spherical in shape. Analysis of 200 nanoparticles yielded an average diameter of 50 ± 18 nm. Following Nafion coating, the nanoparticles appear larger in size (113 ± 35 nm diameter), as shown in Figure 1(c). However, it is important to note that this apparent increase in size could be influenced by the rapid degradation of Nafion under the electron beam, which may obscure smaller particles and affect the accuracy of size measurements. Despite these challenges, the SEM analysis post-Nafion coating confirmed that there were no significant morphological changes, such as agglomeration, and that the Cu_2_O nanoparticles maintained their integrity and remained well adhered to the electrode. Furthermore, no significant changes in the morphology of Cu_2_O were observed after the electrochemical analysis of CH₃OH, both in the absence and presence of Nafion, as illustrated in Figures 1(b) and 1(d), respectively.

**Figure 2:**
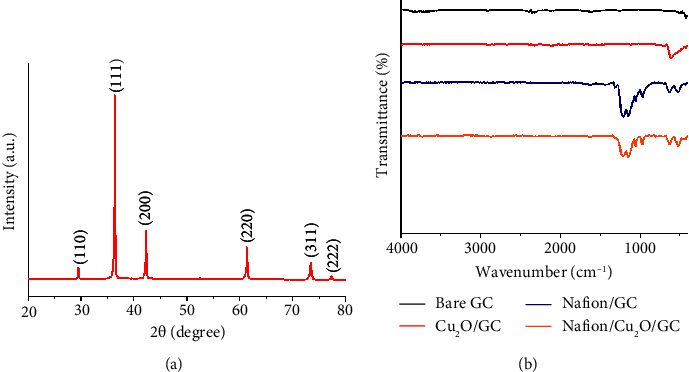
(a) The XRD pattern of Cu_2_O nanoparticles. (b) FTIR spectra of bare GC (black), Cu_2_O/GC (red), Nafion/GC (blue), and Nafion/Cu_2_O/GC (orange).

Figure 2(a) illustrates the X-ray diffraction (XRD) pattern obtained for the Cu_2_O nanopowder. It reveals distinct peaks located at 2*θ* values of 29.54°, 36.40°, 42.28°, 61.51°, 73.49°, and 77.34°, which correspond to the (110), (111), (200), (220), (311), and (222) crystallographic planes, respectively [21]. These peaks conform to established standards (JCPDS no. 5-669) and are consistent with the Pn-3m(224) cubic Cu_2_O space group configuration, characterized by a lattice parameter of 4.27 Å [30, 31].

The surface functional groups of both Cu_2_O and Nafion-coated Cu_2_O nanoparticles immobilized on glassy carbon (GC) substrates were subsequently examined using Fourier transform infrared (FTIR) spectroscopy, as demonstrated in Figure 2(b). The FTIR spectrum of Cu_2_O/GC revealed a prominent peak at approximately 608 cm^−1^, corresponding to the vibration of Cu(I)–O, consistent with previous literature reports [31]. Meanwhile, the FTIR spectrum of Nafion/GC exhibited peaks at 629 cm^−1^ (rocking mode of CF_2_), 968 cm^−1^ (symmetric stretching of C–O–C), 1055 cm^−1^ (symmetric stretching of –SO_3_^–^), 1149 (symmetric stretching of CF_2_), and 1211 cm^−1^ (asymmetric stretching of CF_2_) [32, 33]. For the Nafion/Cu_2_O/GC, the FTIR peaks were observed at 628 cm^−1^ (rocking mode of CF_2_), 969 cm^−1^ (symmetric stretching of C–O–C), 1056 cm^−1^ (symmetric stretching of –SO_3_^–^), 1149 (symmetric stretching of CF_2_), and 1213 cm^−1^ (asymmetric stretching of CF_2_). It is noted that the responses of Cu(I)–O at 608 cm^−1^ overlap with the Nafion responses at 629 cm^−1^.

### 3.2. Voltammetry of CH_3_OH at GC, Cu_2_O/GC, Nafion/GC, and Nafion/Cu_2_O/GC Electrodes

The electrochemical properties of CH_3_OH were investigated at different electrode materials, including glassy carbon (GC), copper(I) oxide nanoparticle-modified glassy carbon (Cu_2_O/GC), Nafion-modified glassy carbon (Nafion/GC), and Nafion-coated copper(I) oxide nanoparticle-modified glassy carbon (Nafion/Cu_2_O/GC) electrodes.

**Figure 3:**
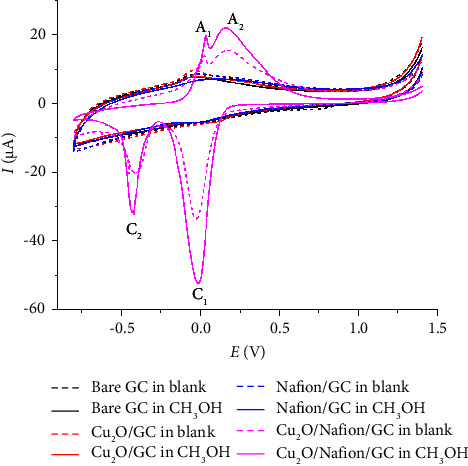
CV of 200 mM CH_3_OH in 0.10 M KCl (solid lines) vs. blank 0.10 M KCl (dashed lines) at bare GC (black), Cu_2_O/GC (red), Nafion/GC (blue), and Nafion/Cu_2_O/GC (pink) at a scan rate of 50 mV·s^−1^.

At the bare GC, Cu_2_O/GC, and Nafion/GC electrodes, neither oxidative nor reductive response was observed in both the absence and presence of CH_3_OH, indicative of sluggish electron transfer kinetics, as depicted in Figure 3. In contrast, distinct voltammetric peaks were evident at the Nafion/Cu_2_O/GC electrode under similar conditions. Specifically, in a blank 0.10 M KCl electrolyte, the Nafion/Cu_2_O/GC electrode displayed two reduction peaks at −0.069 V (designated as C_1_) and −0.429 V (designated as C_2_), and two oxidation peaks at 0.037 V (designated as A_1_) and 0.169 V (designated as A_2_) as shown in Figure 3.

The reduction of Cu_2_O at peak C_1_ is described by the following reaction [21].

(1) $$\begin{array}{c} {\text{Cu}}_{2}O\left(s\right)+H_{2}O\left(l\right)+2e^{‒}⟶2\text{Cu}\left(s\right)+2{\text{OH}}^{‒}\left(\text{aq}\right). \end{array}$$

It was observed that only a minor portion (less than 1%) of the Cu_2_O deposited undergoes reduction, even with Nafion present [21]. This limited reduction is attributed to two primary factors: firstly, the large particle size and the tendency of these particles to aggregate, reducing effective contact with the electrode and thus impeding electron transfer [34]. Secondly, the build-up of hydroxide ions at the electrode following Cu_2_O reduction can slow down the electron transfer kinetics. This accumulation potentially facilitates the formation of CuOH, thereby inhibiting further reduction of Cu_2_O [21].

As the cyclic voltammetry was extended to more negative potentials, an additional reduction peak, labeled C_2_, emerged. This peak is most plausibly attributed to the adsorption of hydrogen, as described in the following reaction [35, 36]:

(2) $$\begin{array}{c} \text{Cu}\left(s\right)+H^{+}\left(\text{aq}\right)+e^{‒}⟶\text{Cu}-H_{\text{ads}}. \end{array}$$

Hydrogen adsorption peaks have been noted in previous studies; typically, these peaks are minimal in the initial voltammetric cycle and become more pronounced in subsequent cycles [35, 36]. Nafion's strong proton conductivity likely contributes to a higher availability of H⁺ at the electrode surface, allowing for more efficient hydrogen adsorption. The increased H⁺ mobility facilitated by Nafion also reduces the build-up of OH^‒^ at the electrode, enhancing the H⁺-coupled electron transfer processes. As a result, the Nafion/Cu_2_O/GC electrode exhibits enhanced sensitivity and faster kinetics in reactions involving proton transfer, including both the reduction of Cu_2_O (C_1_) and hydrogen adsorption (C_2_). In addition, the oxidation peaks A_1_ and A_2_ observed in the voltammogram are likely attributable to the reversed oxidation processes corresponding to reactions 1 and 2, respectively.

Although it could be argued that the two reduction peaks, C_1_ and C_2_, may correspond to the reduction of CuO impurity to Cu_2_O at C_1_ and further reduction of Cu_2_O to Cu at C_2_, the X-ray diffraction (XRD) analysis confirmed the material was predominantly Cu_2_O with no CuO detected. Any CuO impurity, if present, would be minimal relative to the Cu_2_O phase. Consequently, one would expect peak C_2_ to be more prominent than peak C_1_ if significant CuO was present, which was not observed in our experiments.

Upon the addition of 200 mM CH_3_OH, significant enhancements were observed in both oxidation and reduction peaks, as depicted in Figure 3. These enhancements are largely due to the physical and chemical properties of CH_3_OH. CH_3_OH's relatively low viscosity and dielectric constant compared to H_2_O lead to a reduction in overall hydrogen bonding within the solution. This alteration results in less tightly solvated ions, especially H^+^, thereby increasing their mobility through the solution. Increased H^+^ mobility can promote the electrochemical reactions outlined in equation (1).

Moreover, CH_3_OH may interact with OH^−^ generated during the reduction of Cu_2_O [37]. These interactions, possibly involving weak hydrogen bonding or other transient mechanisms, could reduce the local concentration of OH^−^ near the electrode surface. CH_3_OH might also temporarily adsorb onto the electrode surfaces due to dipole interactions or Van der Waals forces, leading to subtle changes in surface wetting or reactant orientation [15].

In the presence of Nafion, which contains negatively charged SO_3_^–^ groups, the role of CH_3_OH becomes even more critical. As a polar molecule, CH_3_OH can facilitate the movement of H^+^ through the Nafion membrane, thus enhancing H^+^ mobility. As previously mentioned, this increase in local H^+^ concentration at the electrode surface is crucial for the reduction of Cu_2_O as it supports the neutralization process during electroreduction. It helps prevent the buildup of OH^−^ at the electrode, which could otherwise lead to the formation of CuOH [21]. This synergistic interaction effectively boosts the electroreduction efficiency of Cu_2_O. It is therefore evident that the utilization of the Nafion/Cu_2_O/GC electrode can be used in the electrochemical analysis of CH_3_OH. The electrochemical characteristics of the voltammetric peaks are further investigated in detail in the following sections.

### 3.3. Tafel Analysis and Effect of Scan Rates

The cyclic voltammetry of CH_3_OH at a Nafion/Cu_2_O/GC electrode, conducted at a slow scan rate of 10 mV·s^−1^, was subjected to Tafel analysis (equation (1) [38]. The analysis of the first reduction peak (C_1_) yielded a value of *n*′+*α*_*n*′+1_ of 0.49 ± 0.01. These findings suggest that the first electron transfer serves as the rate-determining step (*n*′ = 0). The cathodic transfer coefficient of this pivotal electron transfer step (*α*_*n*′+1_) was determined to be 0.49 ± 0.01.

(3) $$\begin{array}{c} \frac{\partial \ln\;I}{\partial E}=\frac{\left(n^{\prime }+\alpha_{n^{\prime }+1}\right)F}{\text{RT}}, \end{array}$$

where *I* is the measured current, *E* is the applied potential, *n*′ is the number of electrons transferred before the rate-determining step, *α*_*n*′+1_ is the cathodic transfer coefficient of the rate-determining electron transfer step, *F* is the Faraday constant (96,485 C·mol^−1^), *R* is the molar gas constant (8.314 J·K^−1^·mol^−1^), and *T* is the temperature (*K*). Note that the Tafel analysis only took into account currents within the 15% to 50% range of peak currents. This approach aimed to minimize the effects of background capacitive currents and diffusional mass transport in the evaluation of electron transfer kinetics [39, 40].

**Figure 4:**
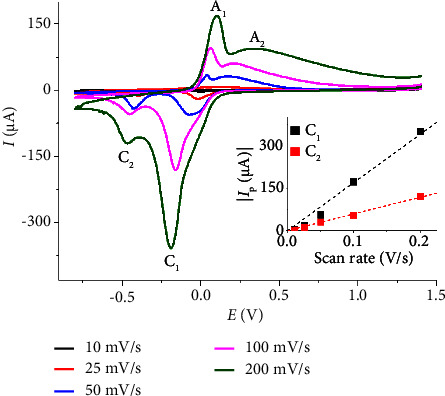
CV of 200 mM CH_3_OH in 0.10 M KCl at a Nafion/Cu_2_O/GC electrode at different scan rates (10–200 mV·s^−1^). The inlay shows the plots of C_1_ and C_2_ peak currents against scan rates.

Figure 4 illustrates the voltammograms obtained from a 200 mM CH_3_OH solution in 0.10 M KCl, using a Nafion/Cu_2_O/GC electrode at varying scan rates. The currents associated with both the first and second reduction peak currents show linear dependence on the scan rates, indicative of adsorption-controlled or surface-confined electrochemical processes. Notably, the ratio of *I*_p,C1_ to *I*_p,C2_ increases with the scan rate. This behavior implies that the reactions corresponding to the C_1_ peak (equation (1)) may feature higher reversibility or faster electron transfer kinetics than the C_2_ peak (equation (2)), allowing the peak current for these reactions to scale more effectively with increasing scan rates.

### 3.4. Effects of the Amount of Cu_2_O Nanoparticles

**Figure 5:**
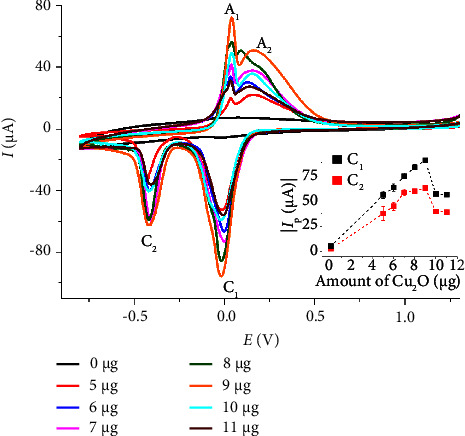
CV of 200 mM CH_3_OH in 0.10 M KCl at Nafion/Cu_2_O/GC electrodes prepared using different amounts of Cu_2_O nanoparticles at a scan rate of 50 mV·s^−1^. The inlay shows the plots of C_1_ and C_2_ peak currents against the amount of immobilized Cu_2_O nanoparticles.

Figure 5 elucidates the influence of surface-deposited Cu_2_O nanoparticle quantity on CH_3_OH analysis. As the amount of Cu_2_O nanoparticles increases, all peaks exhibit a concomitant rise, confirming their association with Cu_2_O. These nanoparticles provide active sites for the redox reactions and increase the electrode's effective surface area, thereby increasing the Faradaic currents. However, beyond 9.0 *μ*g of Cu_2_O nanoparticles, the voltammetric peak currents no longer increase with additional Cu_2_O. This diminution may be ascribed to the formation of multilayers of Cu_2_O nanoparticles, hindering the mass transport of CH_3_OH and other relevant species.

Another significant phenomenon is the overlapping of diffusion zones surrounding individual Cu_2_O nanoparticles. At lower nanoparticle concentrations, each nanoparticle has its own distinct diffusion layer, where radial diffusion of relevant species such as CH_3_OH occurs efficiently. However, as the nanoparticle density increases, the diffusion zones begin to overlap, leading to a transition from radial diffusion to more planar, linear diffusion across the entire electrode surface. This shift results in diminished current per nanoparticle, as the benefits of convergent diffusion around isolated nanoparticles are lost, reducing the contribution of individual nanoparticles to the overall current [41–44].

Furthermore, nanoparticle aggregation can exacerbate this issue. As nanoparticles aggregate, their effective surface area decreases, and some nanoparticles may become inactive or less accessible due to entrapment within aggregates. This aggregation not only reduces the number of available active sites but also increases resistance to charge transfer, further reducing the electrode's performance [45]. Consequently, 9.0 *μ*g of Cu_2_O nanoparticles was chosen as the optimal amount for the CH_3_OH analysis in subsequent sections.

### 3.5. Calibration Plot and Reproducibility Test

**Figure 6:**
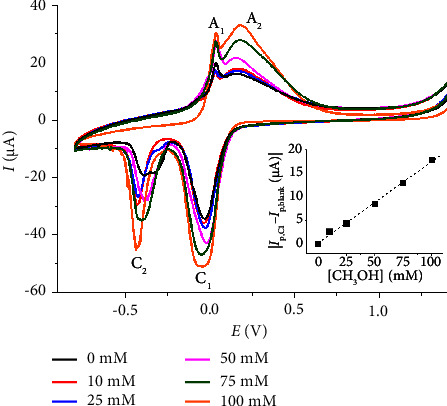
CV of varied concentrations of CH_3_OH in 0.10 M KCl at a Nafion/Cu_2_O/GC electrode at a scan rate of 50 mV·s^−1^. The inlay shows the calibration plot of C_1_ peak currents against CH_3_OH concentrations.

Figure 6 illustrates the voltammograms obtained from different CH_3_OH concentrations at a Nafion/Cu_2_O/GC electrode. The C_1_ reduction peak currents demonstrated a linear increase within the range of 0.33–100 mM CH_3_OH, with a sensitivity of 0.17 *μ*A·mM^−1^. The limit of detection (3 s/m) was determined to be 0.10 mM. Measurements using peak C_1_ conducted with three different Nafion/Cu_2_O/GC electrodes demonstrated relative standard deviations of 1.1%, 1.7%, and 2.6% at CH_3_OH concentrations of 10 mM, 50 mM, and 100 mM, respectively, indicating the excellent reproducibility of the sensor.

### 3.6. Selectivity Studies

The selectivity of the Nafion/Cu_2_O/GC sensor for CH_3_OH detection was assessed against potential interferences, including ethanol (C_2_H_5_OH), isopropanol (C_3_H_7_OH), and ethylene glycol ((CH_2_OH)_2_). The results demonstrated that the C_1_ peak current of the Nafion/Cu_2_O/GC sensor remained unaffected by the presence of one-fold concentrations of ethanol, isopropanol, and ethylene glycol, with an experimental error within a 5% relative standard deviation (RSD). This observation indicates the excellent selectivity of the developed sensor. Such selectivity arises from the absorption of alcohols via solvation of the sulfonic acid groups, akin to the interaction between water and Nafion. Notably, larger alcohol molecules diffuse more slowly in the hydrophilic channels of Nafion compared to the smaller methanol and water molecules [46].

### 3.7. Recovery Studies in Hand Sanitizer Spray Samples

The developed Nafion/Cu_2_O/GC sensor was validated using spiking and recovery tests, employing the standard addition method, on hand sanitizer spray samples from three different brands. The results, summarized in Table 1, showed percentage recoveries in the range of 95–107%, indicating the sensor's capability to accurately analyze CH_3_OH concentrations in these matrices.

The analytical performance of the developed Nafion/Cu_2_O/GC sensor is summarized and compared with other existing electrochemical sensors in Table 2. Overall, our sensor exhibits a competitive detection limit and a broad linear range when compared to other electrochemical sensors. Compared to traditional analytical methods such as gas chromatography (GC) [23, 24] and high performance liquid chromatography (HPLC) [25], the Nafion/Cu_2_O/GC sensor provides a more cost-effective and simpler alternative for CH_3_OH detection. While GC and HPLC are renowned for their lower detection limits and comprehensive analytical capabilities, they necessitate extensive sample preparation, sophisticated instrumentation, and specialized operators. In contrast, the Nafion/Cu_2_O/GC sensor enables rapid, on-site testing without the need for extensive setup or specialized training, offering significant advantages in terms of accessibility and ease of use.

### 3.8. Application in the Evaluation of CH_3_OH Adsorption Isotherm

The developed sensor was next applied in the evaluation of CH_3_OH adsorption on activated carbon, one of the most commonly used sorbent materials. The concentration of residual CH_3_OH in the solution was quantified using a Nafion/Cu_2_O/GC electrode via cyclic voltammetry at a scan rate of 50 mV·s^−1^ in the presence of 0.10 M KCl supporting electrolyte. The amounts of CH_3_OH adsorbed on activated carbon were then evaluated by subtracting the residual amount from the initially added amount.

**Figure 7:**
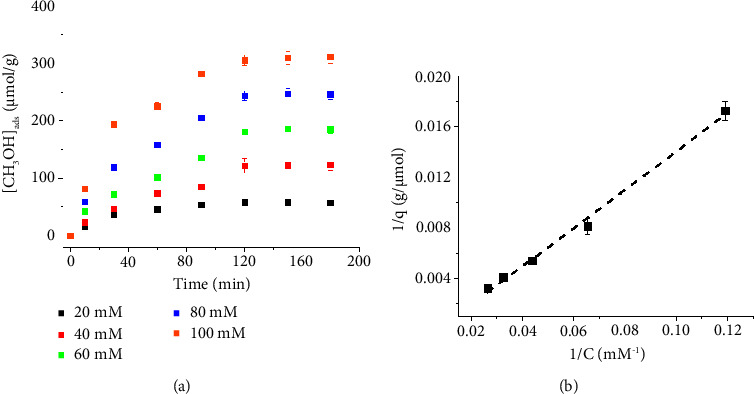
(a) The amounts of CH_3_OH adsorbed on activated carbon at different times for various initial concentrations of CH_3_OH. (b) A plot of 1/*q* against 1/*C* according to the Langmuir model.

Figure 7(a) demonstrates the effects of increasing the adsorption time and initial CH_3_OH concentrations on the adsorption process. After activated carbon was in contact with CH_3_OH for sufficiently long time, adsorption equilibrium was established (CH_3_OH(ads) ⇄ CH_3_OH(aq)). The analyses of CH_3_OH(ads) at equilibrium for different initial CH_3_OH concentrations revealed the adsorption isotherm. The observed experimental data showed that the adsorption of CH_3_OH on activated carbon followed the Langmuir adsorption model (equation (4)).

The linear form of the Langmuir isotherm is given by the following equation [54]:

(4) $$\begin{array}{c} \frac{1}{q}=\left(\frac{1}{Bq_{\max}}\right)\left(\frac{1}{C}\right)+\frac{1}{q_{\max}}, \end{array}$$

where *B* is the equilibrium constant (= rate constant of adsorption/rate constant of desorption), *C* is the initial CH_3_OH concentration added to the solution, *q* is the amount of CH_3_OH adsorbed on activated carbon at equilibrium, and *q*_max_ is the maximum adsorption capacity of the monolayer.

The results thus suggested that the adsorption of CH_3_OH on activated carbon is the monolayer, and that a fraction of the available uniform adsorption sites was occupied randomly with no interaction between adjacent sites. The maximum monolayer adsorption capacity of CH_3_OH on activated carbon was determined to be 609 ± 62 *µ*mol·g^−1^. This section therefore demonstrates the advantages of the developed Nafion/Cu_2_O/GC sensor in the fast evaluation of CH_3_OH during the adsorption isotherm studies.

## 4. Conclusions

Through a detailed analysis of its physicochemical and electrochemical properties, this work has developed a Nafion-coated Cu_2_O nanoparticle (Nafion/Cu_2_O/GC) sensor with enhanced electrochemical capabilities for the analysis of methanol. The sensor demonstrated excellent analytical performance, particularly with high selectivity towards methanol. This selectivity is attributed to the selective absorption and diffusion of methanol in the hydrophilic channels of the Nafion membrane, while Cu_2_O acts as the catalytic electrode. Comprising commercially available components, the developed sensor shows promise for future scaling into mass production, offering rapid detection capabilities at low cost. The final tests, which involved detecting methanol in hand sanitizer samples and evaluating of the methanol adsorption process, further confirmed the sensor's utility and effectiveness in public health and safety applications, especially considering the growing importance of methanol monitoring due to its toxicological implications. In addition, the electrochemical processes of methanol at the copper-based electrode could also be useful in developing methanol fuel cells and other relevant methanol-based electrochemical applications.

## Figures and Tables

**Table 1:** CH_3_OH recovery tests in hand sanitizer samples using the Nafion/Cu_2_O/GC sensor.

Hand sanitizer samples	Added CH_3_OH (mM)	Detected CH_3_OH (mM)	Recovery (%)	RSD (%)
Sample 1	10.0	10.4	104	5
Sample 2	10.0	10.7	107	6
Sample 3	10.0	9.5	95	3

**Table 2:** Comparison of electrochemical sensors for CH_3_OH detection.

Electrode	Method	Linear range (mM)	Detection limit (mM)	Ref
Nafion/PtZONS/GC	AMP	0.03–1	0.03	[47]
NiOOH/PANI-Gr	AMP	1–20 and 20–100	0.290	[48]
NiOOH/PANI-CNTs	AMP	1–20 and 20–500	0.323	[48]
PdNPs@SBA-15- Nafion/GC	AMP	0.02–1	0.012	[49]
SE/PtNPs/GC	AMP	0.25–10 and 50–10000	0.1	[50]
*α*-Fe_2_O_3_/SnO_2_NCs/Ag	LSV	0.25–250	0.16	[51]
Au-decorated carbon nanocomposite	LSV	1–80	0.39	[52]
Polythiophene/*α*-Fe_2_O_3_	LSV	5–1000	1.59	[53]
Nafion/Cu_2_O/GC	CV	0.33–100	0.10	This work

AMP: amperometry; CNTs: carbon nanotubes; CV: cyclic voltammetry; GC: glassy carbon; Gr: graphene; NCs: nanocubes; NPs: nanoparticles; PANI: polyaniline; PtZONS: platinum nanoparticle-incorporated ZnO hybrid nanospheres; SBA-15: Santa Barbara amorphous-15-PrNHEtNH_2_; SE: silicon epoxy.

## Data Availability

The data used to support the findings of this study are available from the authors on request.
